# A Hospital-Community-Family–Based Telehealth Program for Patients With Chronic Heart Failure: Single-Arm, Prospective Feasibility Study

**DOI:** 10.2196/13229

**Published:** 2019-12-13

**Authors:** Xiaorong Guo, Xiang Gu, Jiang Jiang, Hongxiao Li, Ruoyu Duan, Yi Zhang, Lei Sun, Zhengyu Bao, Jianhua Shen, Fukun Chen

**Affiliations:** 1 Clinical Medical College Yangzhou University Yangzhou, Jiangsu China; 2 Dalian Medical University Dalian, Liaoning China; 3 Department of Cardiology Subei People’s Hospital Yangzhou, Jiangsu China

**Keywords:** telehealth, chronic heart failure, feasibility studies, precise follow-up, self-management

## Abstract

**Background:**

An increasing number of patients with chronic heart failure (CHF) are demanding more convenient and efficient modern health care systems, especially in remote areas away from central cities. Telehealth is receiving increasing attention, which may be useful to patients with CHF.

**Objective:**

This study aimed to evaluate the feasibility of a hospital-community-family (HCF)–based telehealth program, which was designed to implement remote hierarchical management in patients with CHF.

**Methods:**

This was a single-arm prospective study in which 70 patients with CHF participated in the HCF-based telehealth program for remote intervention for at least 4 months. The participants were recruited from the clinic and educated on the use of smart health tracking devices and mobile apps to collect and manually upload comprehensive data elements related to the risk of CHF self-care management. They were also instructed on how to use the remote platform and mobile app to send text messages, check notifications, and open video channels. The general practitioners viewed the index of each participant on the mobile app and provided primary care periodically, and cardiologists in the regional central hospital offered remote guidance, if necessary. The assessed outcomes included accomplishments of the program, usability and satisfaction, engagement with the intervention, and changes of heart failure–related health behaviors.

**Results:**

As of February 2018, a total of 66 individuals, aged 40-79 years, completed the 4-month study. Throughout the study period, 294 electronic medical records were formed on the remote monitoring service platform. In addition, a total of 89 remote consultations and 196 remote ward rounds were conducted. Participants indicated that they were generally satisfied with the intervention for its ease of use and usefulness. More than 91% (21/23) of physicians believed the program was effective, and 87% (20/23) of physicians stated that their professional knowledge could always be refreshed and enhanced through a library hosted on the platform and remote consultation. More than 60% (40/66) of participants showed good adherence to the care plan in the study period, and 79% (52/66) of patients maintained a consistent pattern of reporting and viewing their data over the course of the 4-month follow-up period. The program showed a positive effect on self-management for patients (healthy diet: *P*=.046, more fruit and vegetable intake: *P*=.02, weight monitoring: *P*=.002, blood pressure: *P*<.001, correct time: *P*=.049, and daily dosages of medicine taken: *P*=.006).

**Conclusions:**

The HCF-based telehealth program is feasible and provided researchers with evidence of remote hierarchical management for patients with CHF, which can enhance participants’ and their families’ access and motivation to engage in self-management. Further prospective studies with a larger sample size are necessary to confirm the program’s effectiveness.

## Introduction

### Background

Chronic heart failure (CHF) is a major public health issue, affecting nearly 1 in every 100 people aged above than 65 years, and its prevalence is increasing with the aging population [1]. Patients with CHF have a poor quality of life and a very low 5-year survival rate [2]. In addition, they are particularly vulnerable to readmission. Previous studies indicated that the 30-day readmission rate was 5.6% for CHF, and more than half of them were readmitted a year later [3,4]. In addition to the morbidity of repeat hospitalizations, the cost of hospitalizations is high [5,6]. It undoubtedly increases the burden on the health care system and results in unnecessary wastage of medical resources.

Considering the demographic changes of patients with CHF, there is a need for a solution that can facilitate more convenient and effective access to medical service, especially in remote areas. With ubiquitous penetration of wireless internet, mobile phone, and portable personal health tracking devices, telehealth has become possible. On account of its potential to provide a much larger number of patients across a much greater geography with specialist care, it is increasingly being valued as a promising long-term management strategy for patients with chronic diseases. It promises the opportunity to *remotely* provide patients with consistent education, motivation to become engaged in their own self-care, and assistance in monitoring on a regular basis [7].

To date, many experiments have confirmed the remarkable achievements of telehealth in decreasing patients’ risk of CHF exacerbations and hospital readmissions [8-11], which addresses the burdens associated with disease management and reduces CHF-associated health care costs [12]. However, a number of prior research studies have selected general practitioners or cardiologists to be the only management lead [13,14] or adopted a single management strategy to support self-care and home telemonitoring, such as structured telephone programs [10,15], mobile apps [11], or Web platforms [16]. To our knowledge, no existing system has incorporated a Web platform, mobile app, and smart health tracking devices, together with collaborative work between general practitioners and cardiologists, to engage and empower patients in disease self-management.

### Objectives

We developed a hospital-community-family (HCF)–based telehealth program and aimed to explore its feasibility for the implementation of remote hierarchical management in patients with CHF.

## Methods

### Innovative Digital Devices in the Hospital-Community-Family–Based Telehealth Program

Overall, the program consisted of three innovative digital devices: (1) a remote monitoring service platform (Physio-Gate PG 1000, GETEMED Medizin- und Informationstechnik AG) that collects and integrates patients’ data, (2) a personal health tracking mobile app (King OPTO-Electronic) for each patient, and (3) a few smart health tracking devices.

#### The Integrated Remote Monitoring Service Platform

The integrated remote monitoring service platform is a cloud-based, tablet computer–accessed, secure Web platform that was designed by the Information Academy of Yangzhou University to collect and integrate data. Its domain name is iccvd.com. It is available for both doctors and participants. Each doctor has his/her own specific log-in account. Participants can browse the Web directly without logging in. In the HCF-based telehealth program, this platform acted as a medium for information transmission and sharing of resources among regional central hospitals, community hospitals, and patients. The main functions of this platform are as follows:

- The electronic medical record of each participant, including medical history, physical examinations, laboratory and imaging findings, clinical diagnosis, general treatment, medication, and individualized reminder, was recorded on the remote monitoring service platform. There are three different important data sources: (1) data registered at the clinic, (2) data exported from the hospital information system, and (3) data recorded by participants themselves.
- In the electronic medical record, previous data about laboratory and imaging findings were cumulated and arranged in a chronological order. Each value was color marked to indicate the health status of participants: green for improvement, orange for medium risk, red for high risk, and no color for normal or close to normal range. Thus, doctors can assess a patient’s condition more quickly and comprehensively from high-to-moderate risk to low risk, as all medical information is available at a glance.
- What makes the platform different is the function of analyzing the cumulative data of each individual. A trend diagram of quantitative data such as blood pressure (BP), heart rate, and 6-minute walk distance can be generated automatically by the platform, which can help visualize the overall dynamic change of data.
- The platform, equipped with a dedicated audio-video system, has high confidentiality and anti-interference ability, which is different from public video-audio platforms such as QQ or WeChat (Tencent, Shenzhen, China).
- The platform was designed as a library about CHF, including the current research state, latest developments, future trends, and most influential technologies and theories, with more advanced medical knowledge browsing links to the MedSci medical website.

#### Mobile App

A Web-based app, created on both Android and iOS platforms, was developed for reporting health information, monitoring clinical signs, and enabling direct supervision and instruction for patients. The app was designed with a link to the remote monitoring service platform, and two versions were available: one for physicians and another for patients. Both participants and physicians have the right to use the mobile app free of charge. It enables participant management from a distance and allows the physicians to observe and follow the health status of participants at any time.

**Figure 1:**
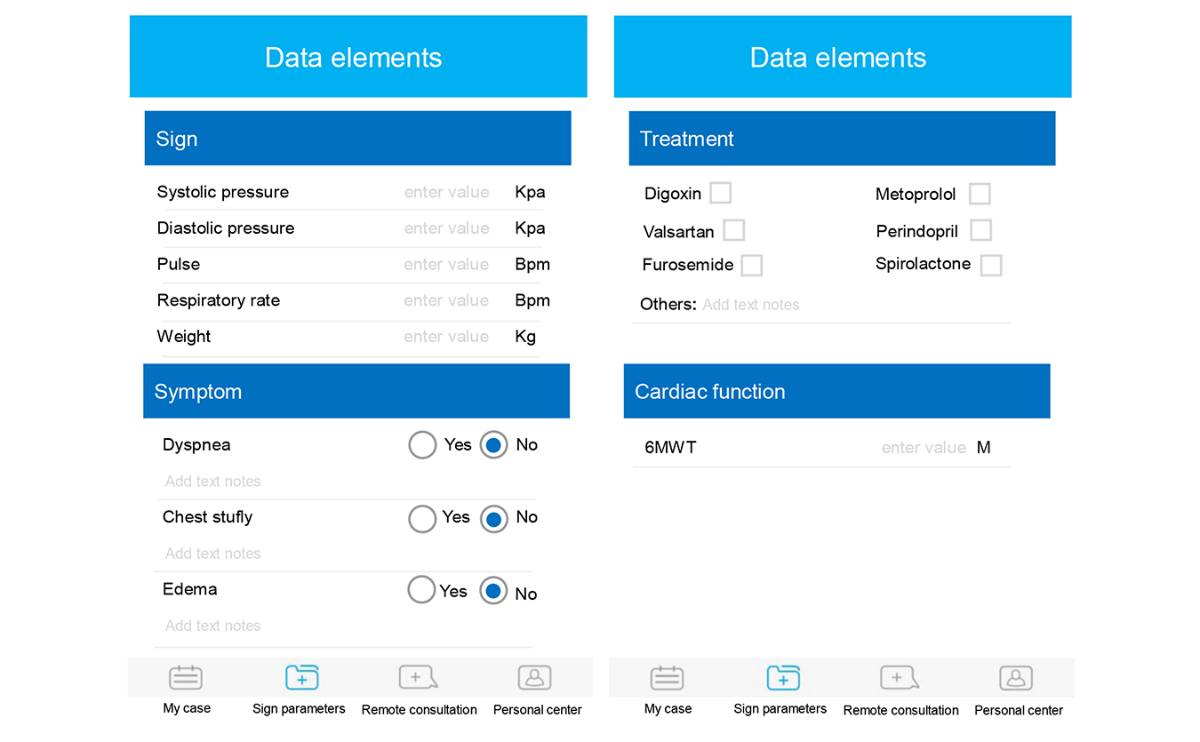
Screenshots from the mobile app of patient.

The patient’s mobile app is secured by their own specific password. It has the following main functions: data uploading, remote consultations, electronic medical record viewing, and medical appointments (Figure 1). Participants can use the app at home to record and upload comprehensive data elements related to the risk of CHF self-care management, including daily recording of symptom and sign changes and medication adherence. Physicians will analyze these incoming values. When data are outside an acceptable range, participants will receive video calls or text messages via the app. In addition, participants can communicate with physicians more conveniently through their handheld mobile phone with the assistance of the remote consultations function of our app. They can send text messages to their general practitioners round the clock for help and receive personalized guidance on long-term self-care management of CHF via the mobile app. Moreover, participants and their family members can view the participant’s cumulative data, including examination report and clinical diagnosis and medication. Finally, reminders for tracking medical appointments and next visits are visible on the mobile app to keep track of their schedule.

A mobile app for physicians was also designed. When a participant uploads data or clicks on the remote consultation icon, general practitioners receive text messages that alert them. They can then view the electronic medical record of the participant under their jurisdiction at any time by logging into the mobile app. If necessary, they can send text or video messages to participants and provide them with individualized advice through mobile apps.

#### Smart Health Tracking Devices

**Figure 2:**
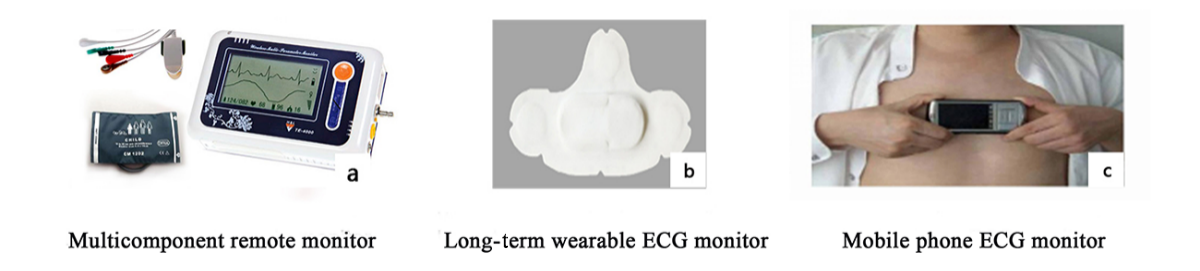
Smart health tracking devices. ECG: electrocardiogram.

The basic devices including a weight scale and a BP monitor with a cuff were prepared by participants themselves. Some smart health tracking devices are available in our program, if necessary: multicomponent remote monitor (for an electrocardiogram [ECG], peripheral capillary oxygen saturation, and BP; hlTE-4000X, TE-4000Y, Beijing Haileying Medical Technology Co, Ltd, Beijing, China), long-term wearable ECG monitor (BECG1200-A, Thoth Medical Technology Co, Ltd, Suzhou, China), and mobile phone ECG monitor (Zhongwei Laikang Technology Development Co, Ltd, Beijing, China; Figure 2). These health tracking devices were invented to allow remote monitoring, recording, transmitting, and analysis of health parameters during daily activities.

### Study Design

#### Population

This single-arm, prospective, quasi-experimental study was conducted in the midland of Jiangsu province. The study received approval from the institutional review board of Subei People’s Hospital. Patients were recruited consecutively from the outpatient clinics of our medical center between June 2017 and September 2017 and were followed up for at least 4 months.

#### Inclusion Criteria

The inclusion criteria were as follows: (1) age ≥18 years; (2) left ventricular ejection fraction of ≤45% as assessed by echocardiogram, but left ventricular ejection fraction can be >45% for patients with cardiac insufficiency caused by atrial fibrillation, valvular heart disease, and hypertrophic cardiomyopathy; (3) history of CHF of ≥3 months with New York Heart Association functional class I to IV, of which class IV was without strict bed rest; (4) the general intervention rules are based on the current guidelines [5,17]; and (5) ability to understand the requirements of the study and will to provide written informed consent.

#### Exclusion Criteria

The exclusion criteria were as follows: (1) some secondary cardiomyopathy (eg, hyperthyroid heart disease, anemic heart disease); (2) a history of malignancy and life expectancy <1 year; (3) severe primary hepatic and renal insufficiency (alanine aminotransferase level ≥ 100 U/L, serum creatinine level > 3.0 mg/dL, and serum albumin level < 2.5g/L); (4) refusal to participate; (5) inability to visit outpatient clinics periodically; (6) ambulatory status; and (7) judged to be inappropriate for the study by the researchers.

### Study Procedure

General practitioners in 12 peripheral community hospitals who agreed to participate and signed the contract were organized and trained about the study procedures, the latest standardized management, and the treatment of CHF before the trial began.

On the patient’s first outpatient visit, the investigator introduced the study plan to the patient and his or her caregiver (if present) who met the inclusion and exclusion criteria. The mobile app was downloaded to patient’s iOS or Android mobile phone or tablet device after the procedures were explained, and written informed consent of the patient was obtained. Trained research assistants then instructed the patient on participation of the HCF-based telehealth program (described below) and the use of digital devices. At study enrollment, information on participant demographics was collected, and the first electronic medical record was established on the remote monitoring service platform. Thereafter, participants were assigned to the nearest participating community hospitals for further follow-up and management. Participants then took their health tracking devices and mobile apps home to join our HCF-based telehealth program, in which they were reminded to use the app or browse the Web platform more than once a week. Telephone follow-up was scheduled at weeks 1, 4, 8, and 16 to evaluate and provide technical support regarding the use of digital devices.

**Figure 3:**
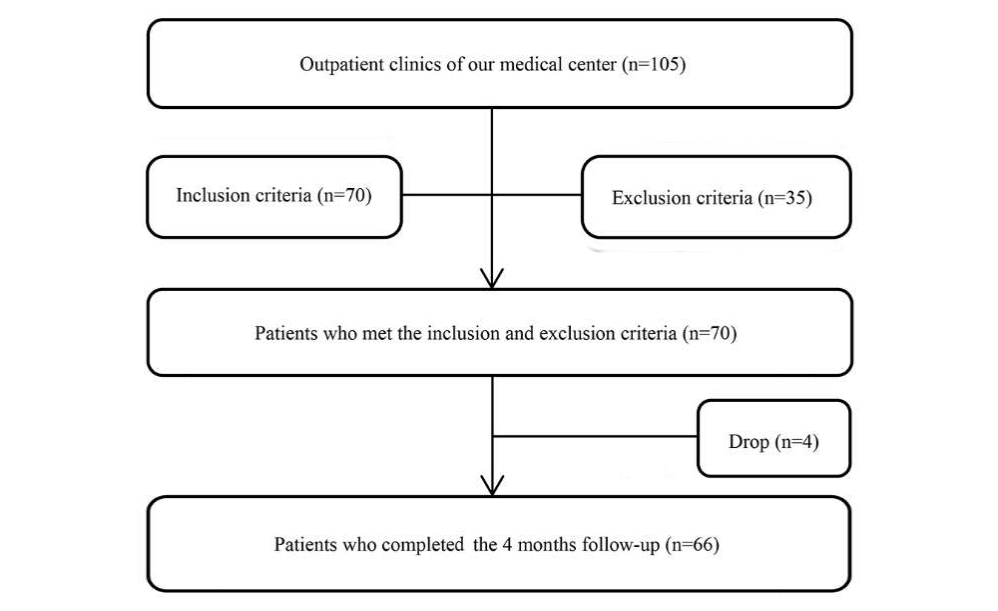
Study flow of HCF-based telehealth program. HCF: hospital-community-family.

At the end of the study, the accomplishments of the HCF-based telehealth program were summarized and engagement in the intervention was assessed. Participants, their family members, and professional health care professionals were also interviewed for their experiences and opinions about the telehealth program. Participants were encouraged to provide their honest and candid feedback about the program. The study flow is illustrated in Figure 3.

### The Hospital-Community-Family–Based Telehealth Program

**Figure 4:**
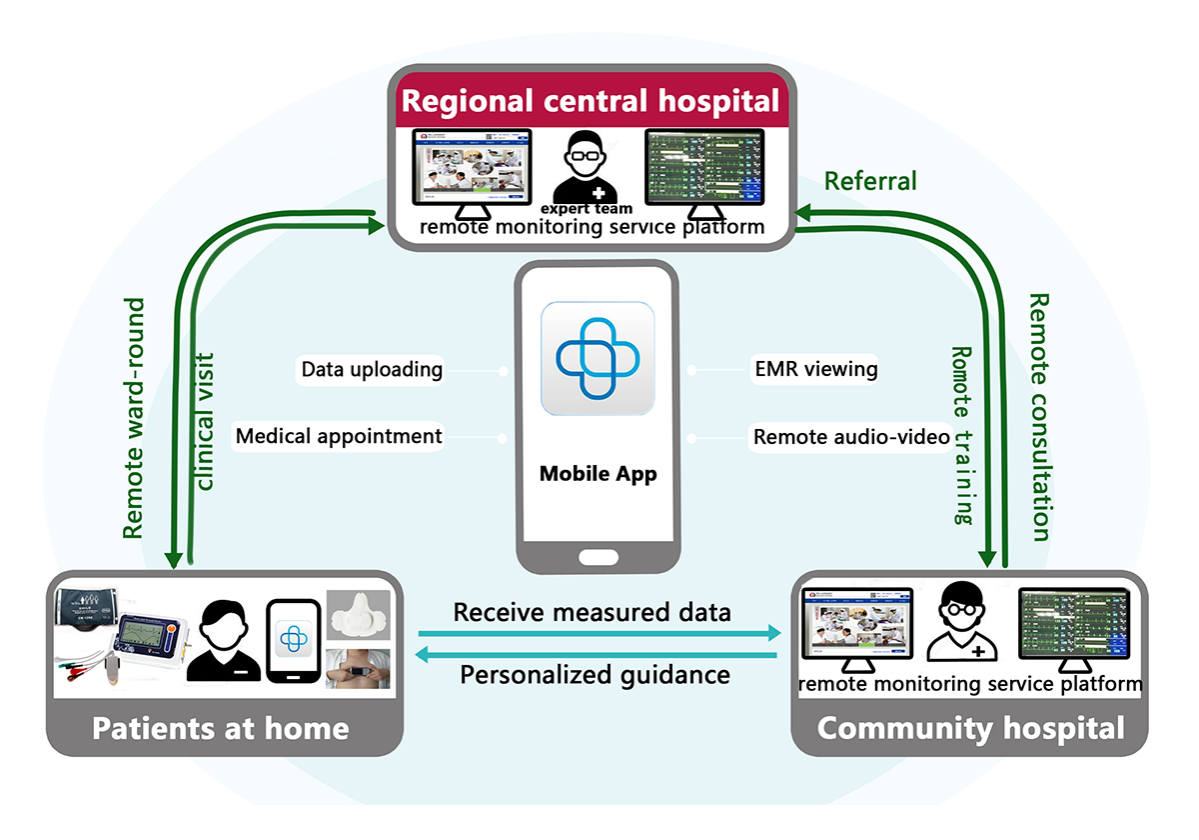
The hospital-community-family–based telehealth program in patients with chronic heart failure. EMR: electronic medical record.

The HCF-based telehealth program consisted of multidisciplinary team members, including experienced cardiologists, general practitioners, participants, and their family members (Figure 4). Mobile app, as the main communication tool between different parts, is at the core of the telehealth structure. The HCF-based telehealth program was not a purely Web-based trial, and there were face-to-face components included in the intervention and assessment.

The program was partly financed by the project of the Jiangsu Provincial Science and Technology Department (project code: BL2013022) for items such as remote equipment, software development, labor subsidies, and academic expenses. The primary care physicians and cardiologists who joined the program were compensated or remunerated according to their workload and contribution to the project.

During the study, participants were required to visit the cardiologist clinic at least once every 2-4 months, and examination of indices related to CHF was arranged every 3-6 months or on the basis of the participant’s condition. During regular clinical visits, participants could provide their health information via the mobile app at home and browse the Web-based platform at any time. As for participants with CHF who were at high risk of arrhythmia, a smart health tracking device was provided to record and send health parameters at any moment. Owing to the remote consultation function of the mobile app, patients could contact with the general practitioner 24 hours a day if they felt uncomfortable. Subsequently, they would receive feedback about their measurements, education about their symptoms and their diseases, reminders, and encouragement to follow care plans [18].

The general practitioners in community hospitals examined the data submitted by the participants and artificially identified abnormal or worsening health data of participants. Depending on the risk level of the participant, the general practitioners would send out a phone confirmation or perform other interventions, such as consulting experienced cardiologists at our regional central hospital. In some cases, the result may be a referral patient to a superior hospital through a *green passage*. Usually, general practitioners would initiate weekly interactive voice calls to assess the health status and provide technical support on the use of digital devices. Changes in the health status of each participant would be reported on the remote monitoring service platform in the electronic medical records form monthly by general practitioners. The comorbidities and medication would be kept up to date, as they were the reasons for adjusting the treatment regimen.

Experienced cardiology specialists in regional central hospitals, through the app’s audio and video system, conducted weekly ward rounds for patients reported to be critically ill by general practitioners, tailored CHF management, and adjusted outpatient visit schedules according to the clinical situation of each participant. In addition, experts regularly held remote video lectures on the treatment and management of CHF and provided free training for general practitioners throughout the study.

A unique feature of this project was the collaborative work between general practitioners and cardiologists using a Web-based platform, a variety of intelligent health tracking devices, and mobile apps to achieve a comprehensive and personalized intervention for patients with CHF.

### Outcome Measures

Usability of the HCF intervention for patients was assessed based on the 12-item Perceived Health Web Site Usability Questionnaire [19], which is a widely used tool to assess usability for a variety of technologies. It consists of three separate components that ask about a patient’s satisfaction with the intervention, the ease of use, and the effectiveness. All items were rated on a 1-7 scale. Responses were averaged for each component and across all items. In this study, the Cronbach alpha for satisfaction, ease of use, usefulness, and overall usability were .81, .74, .82, and .83, respectively. The physician’s satisfaction with the intervention was assessed through self-reported questionnaires.

At the beginning and end of the study, lifestyle and health behaviors of participants were collected by interview, which would be used to assess changes in self-management for participants. Engagement with the intervention was assessed objectively via daily Web portal log-ins and use of the mobile app, including data uploading, remote consultations, and electronic medical record viewing. During the interview, a qualitative method was used to examine perceptions of the intervention components for participants.

### Data Analyses

Descriptive statistics were computed for participants’ characteristics and all outcome variables. Data were presented as numbers and percentages for categorical variables and mean and standard deviations for continuous variables. The Wilcoxon signed-rank tests for nonparametric data were used to assess satisfaction with the HCF intervention. Differences between categorical variables were analyzed by the Chi-square test when *P*<.05 was considered statistically significant.

## Results

### Baseline Characteristics of Participants

As of February 2018, of the 105 patients with CHF assessed for eligibility, 70 subjects met the inclusion criteria and agreed to participate in this study. Of the 70 participants, 4 (6%) dropped out from the study: 2 of them were lost to follow-up and 2 others withdrew voluntarily because they had migrated abroad and were unable to visit outpatient clinics periodically. The final analysis was performed in 66 (94%) participants who completed the baseline and 4-month measurements. All demographic information for participants who completed the study is shown in Table 1.

**Table 1:** Baseline demographic characteristics of the study participants.

Characteristics		Values
Age (years), mean (SD)		69.35 (11.15)
**Sex, n**		
	Male	34
	Female	32
Body mass index (kg/m^2^), mean (SE)		22.17 (1.69)
**Clinical history, n (%)**		
	Hypertension	37 (56)
	Coronary heart disease	11 (17)
	Valvular heart disease	11 (17)
	Atrial fibrillation	32 (48)
	Cardiomyopathy	23 (35)
	Diabetes	22 (33)
**Medications, n (%)**		
	Angiotensin-converting enzyme inhibitors/angiotensin receptor blocker	36 (55)
	Beta blocker	40 (61)
	Diuretic	32 (48)
	Digitalis	21 (32)
	Ivabradine	0 (0)
**Device, n (%)**		
	Cardiac resynchronization therapy	3 (5)
**New York Heart Association, n (%)**		
	I	11 (17)
	II	32 (48)
	III	23 (35)
	IV	0 (0)
**Education, n (%)**		
	Less than high school	51 (77)
	High school degree or more	15 (23)
**Home caregiver, n (%)**		
	Spouse	34 (52)
	Relative	21 (32)
	Other	11 (17)
**Monthly income (CNY $), n (%)**		
	≤400	31 (47)
	400-800	21 (32)
	≥800	14 (21)

### Accomplishments of the Hospital-Community-Family–Based Telehealth Program

**Figure 5:**
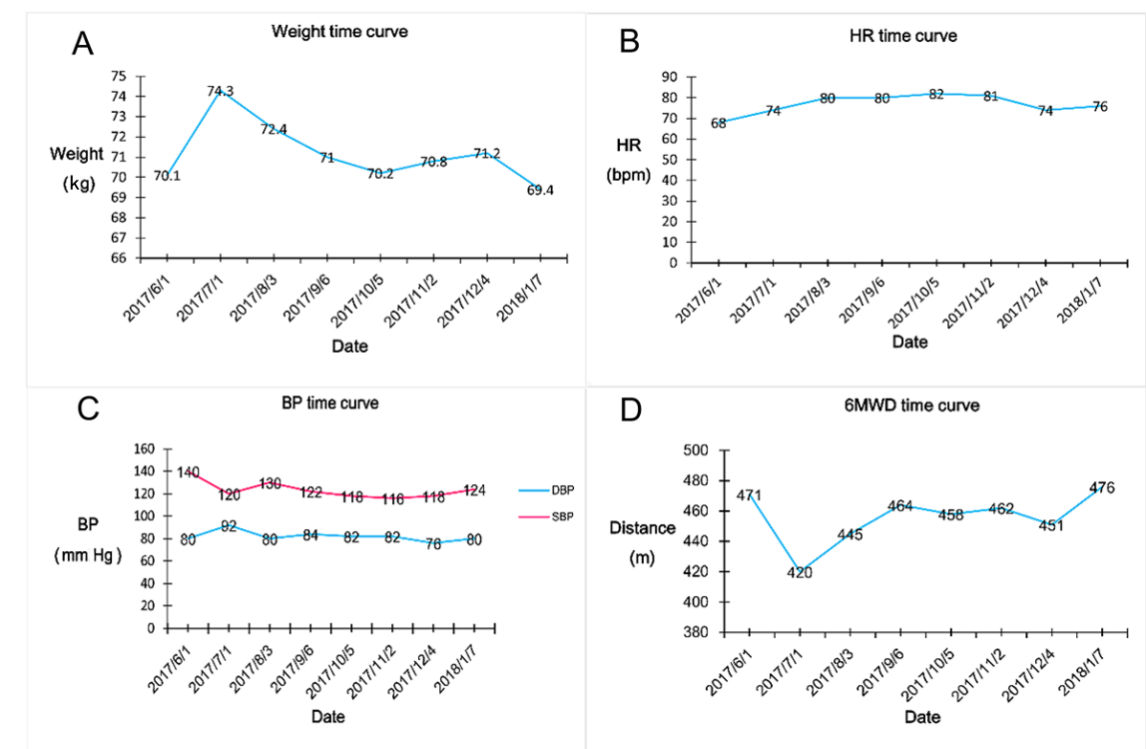
Data trends of a person on the remote monitoring service platform. HR: heart rate; BP: blood pressure; DBP: diastolic blood pressure; SBP: systolic blood pressure; 6MWD: 6-min walk distance.

Participants uploaded data elements recorded by themselves or their caregivers weekly via the mobile app, submitting a total of 1096 reports. Data and the trend diagram of health parameters from one of the participants, analyzed and generated automatically by the platform, is shown in Figure 5. In addition, 294 electronic medical records were recorded on the remote monitoring service platform. Throughout the study period, general practitioners consulted the experienced cardiologists in the regional central hospital remotely 89 times by the mobile app, and one participant was transferred to a superior hospital. Cardiologists completed 196 remote ward rounds and gave advice on adjustments of medications and lifestyle to participants. A total of eight remote interventions failed because of wireless network problems and operational errors by patients.

### Participants and Physicians’ Experience of the Hospital-Community-Family Intervention

As shown in Table 2, the overall usability of the program was slightly above the midpoint of the 1-7 scale. Participants rated their satisfaction with and usefulness of the HCF intervention higher than they rated the ease of use of the HCF intervention.

At the end of the study, 23 physicians, including 18 general practitioners and 5 experienced cardiologists, were surveyed. Results from the survey data showed that 21 (91%) physicians believed that this remote hierarchical management program improves management efficiency, with 20 (87%) physicians stating their professional knowledge can always be refreshed and enhanced through the library hosted on the platform and remote consultation, and all 23 (100%) physicians firmly believed that the program should be explored and promoted on a larger scale.

**Table 2:** Participant-reported usability of hospital-community-family intervention (average scores; possible range=1-7; higher scores indicate greater overall usability, higher degree of satisfaction, easier use, and better effectiveness of the hospital-community-family intervention).

Usability of intervention	Mean (SD)	Range
Overall usability	4.79 (1.03)	2.00-7.00
Satisfaction	5.04 (0.89)	3.00-7.00
Ease of use	4.40 (1.08)	2.00-7.00
Usefulness	4.69 (1.09)	2.00-7.00

### Feedback on Intervention Components

Most participants indicated that they found the mobile app useful as a tool to track CHF-related information, contact clinicians easily, and be reminded to take their medication, and they were also able to share their logged information with the people whom they trust most, such as family members or close friends who could help them during their medical appointments. Participants also responded positively to the knowledge propaganda feature of the remote monitoring service platform, which provided them with useful information, particularly relating to sustaining health. The participants were more interested in smart health tracking devices, which could help them keep track of health conditions anywhere, so that they felt more secure and involved in their own care. Generally, most of them were somewhat or very willing to recommend the telehealth program to patients with CHF around them. We also heard a few complaints about the components that negatively affected their experience. These complaints were mainly focused on the complex operation steps and the consumption of mobile data traffic. Moreover, one patient complained that voice or video call is the largest segment of mobile data traffic unless they can directly connect to the Wi-Fi. Another older patient said that operating this complicated system has more or less obstacles for him.

### Engagement With the Intervention

The overall engagement with the two main components of the intervention (ie, the mobile app and the Web platform) was assessed by participants or their caregivers’ usage logging. More than 60% (40/66) of the study participants showed great adherence to the HCF-based telehealth program, which was defined as the use of the app or the access of Web platform more than once a week and the visit of specialist clinics as scheduled during the study period. Although most of them engaged through the mobile app, 12 participants (18%) who displayed high adherence to the program were found to have used the mobile app and the Web platform simultaneously. In addition, 79% (52/66) of the patients maintained a consistent pattern of reporting and viewing their data over the course of the 4-month follow-up period. We found that overall engagement decreased following the first 8 weeks, but 40 of the 66 participants continued to engage with the care plan throughout each week of the study. Moreover, nearly one-third (21/66) of the participants used a mobile app by themselves, and the rest were mostly family members or caregivers.

### Changes in Lifestyle and Health Behaviors

As shown in Table 3, the program may also have some value in improving health behaviors (increasing fruit and vegetable intake, controlling BP and weight, and reducing salt intake) and drug compliance in participants.

**Table 3:** Changes in lifestyle and health behaviors.

Lifestyle and health behaviors		Baseline, n (%)	At 4 months, n (%)	*P* value
**Diet**				
	Low salt, low fat, and low sugar	37 (56)	48 (73)	.046
	More fruits or vegetables	22 (33)	35 (53)	.02
**Self-monitoring**				
	Blood pressure	15 (23)	41 (62)	<.001
	Weight	5 (8)	19 (29)	.002
**Medicine adherence**				
	Correct daily dosages	41 (62)	55 (83)	.006
	Correct time	35 (53)	46 (70)	.049

## Discussion

### Principal Findings

This is a prospective experimental study to investigate the feasibility of the HCF-based telehealth program in patients with CHF. To the best of our knowledge, this is the first study evaluating the use of such a telehealth program incorporating remote monitoring service platforms, mobile apps, and smart health tracking devices to manage patients with CHF at their own homes. The initial results have been achieved in the program. The results indicate that satisfaction and participation of doctors and patients are relatively higher; meanwhile, the patients’ lifestyle has been effectively improved. Our research is expected to lay a foundation for further large-scale randomized controlled clinical trials related to remote hierarchical medical care.

### Comparison With Prior Work

The main contribution of this program lies in the development of a remote hierarchical management system centered on patients with CHF. This system has multiple intelligent tracking devices that can support patients’ self-care at home and strengthen communications among patients, medical service providers, and home caregivers for better care transition and coordination. To our knowledge, this is one of few systems with collaborative work between general practitioners and cardiologists for HF remote hierarchical management through remote monitoring service platforms, mobile apps, and smart health tracking devices.

This paper presented the design and use of the HCF-based telehealth program for patients with CHF. Similar to other pilot studies [20], we focused on user perceptions and experience because user perspective was the most important dimension in the development phase of telehealth projects [21,22]. According to the interviews of participants, their family members, and physicians, they were generally satisfied with the service. The CHF management team was key in engaging patients to participate the program; the benefits of the program also include support in recording and tracking health status, encouragement and reassurance received from medical staff, timely detection, recognition and management of subtle changes in the condition, and more convenient and faster communication among all participants. Previous investigation showed that physicians did not seem very enthusiastic about telemedicine. The main causes of such opposition were found to be the lack of a medical services delivery system and a professional management team [23]. The HCF-based telehealth program can serve as a platform for providing more continuous care, linking primary and specialty care to support management of patients with severe and chronic diseases. Some physicians perceived the platform to be effective as a communication tool to share data in a timely, accurate, and visual manner, so that they can be armed with all relevant health information contained in one system, especially in an emergency or unfamiliar health care setting, for care planning.

The poor compliance of patients is a major obstacle to the management of most chronic diseases [24,25]. An earlier large trial showed that 14% of the intervention patients never used their equipment, and only 55% were still using the system at the end of the study (6 months from the baseline) [26]. Our most significant finding was that 79% of patients maintained a consistent pattern of reporting and viewing their data over the course of the 4-month follow-up period. In contrast, we found higher engagement levels over the course of the study. A possible explanation for this findings is the user friendliness of digital devices and that family-targeted self-care interventions [27] may play an important role in promoting patient compliance. The caregiver app will show the patient’s real-time data (with the patient’s consent) and receive messages from the patient’s health care providers, so that he/she can help monitor the patient’s health. In fact, telehealth apps have been used to enhance patient-caregiver engagement [28]. Inviting family members or caregivers in training sessions was effective for older adults to continue to adopt the telehealth tools in their daily life. To date, educational interventions intentionally including family involvement in the care of patients with CHF are few, although such family involvement is explicitly recommended in the existing CHF management guidelines [29].

In accordance with reports from the World Health Organization, nearly two-thirds of people’s quality of life and health status lies with their lifestyle and health behaviors, and 53% of the death causes were also associated with lifestyle and personal behaviors. Therefore, we can say that elderly individuals who adopt improved health behaviors would experience a healthy old age [30]. Emerging evidence suggests that telehealth interventions may improve self-care behaviors and disease management for elderly patients [11,31,32]. The Web-based social networks acting as an active communication framework can be an effective means of promoting healthy lifestyles [33]. In the study, patients were encouraged to document their conditions and medicine use and then received personalized health coaching tailored for their individual situation through *face-to-face* communications, which can intensify the patient’s health behaviors, medication adherence, and self-management ability. One possible reason is that the use of mobile health via an app with real-time representation of data trends would strengthen patient empowerment and decision making in self-management. They felt more empowered and confident to perform their self-care activities at home with the help of the program.

### Limitations

This study had some limitations. First, our sample size was small, and the duration of intervention was short. In addition, as this was only a feasibility pilot study, we did not conduct a formal sample size calculation. Furthermore, this was a single-arm, experimental, prospective design study rather than a randomized evaluation and may be not powered to detect the effects of the intervention on clinical outcomes. Large-scale randomized prospective controlled studies will be necessary to test the program. In addition, those who participated in our study were predominantly elderly patients with CHF having varying socioeconomic and educational backgrounds; this limits the generalizability of our findings and precludes us from extrapolating the findings from this study to other populations. Finally, because of technical restriction, semiautomatic input of data in this program greatly wastes human resources and possibly increases the error rate.

### Conclusions

Given the serious and complex condition of patients with CHF, a more convenient and effective access to medical services is urgently needed, especially in remote areas. The HCF-based telehealth program offers them a glimmer of light. On the basis of remote monitoring service platform, mobile app, intelligent health tracking device, and professional management team, this study realized remote hierarchical management of patients with CHF. This study provided evidence on the feasibility of HCF-based telehealth program, potentially enhancing the opportunities and incentives for patients with CHF and their families to participate in self-management. In addition, it may lay the foundation for further large-scale randomized controlled studies. In the near future, we would like to expand the HCF-based telehealth program to other cardiovascular diseases such as atrial fibrillation, coronary heart disease, and stroke.

## Abbreviations

BP: blood pressure
CHF: chronic heart failure
ECG: electrocardiogram
HCF: hospital-community-family
